# Supplementary material for: Population diversity of cassava mosaic begomoviruses increases over the course of serial vegetative propagation
Source: J Gen Virol. 2021 Jul 26;102(7):001622. doi: 10.1099/jgv.0.001622 (PMC8491896; doi:10.1099/jgv.0.001622)
Supplement: Supplementary material 1 [file jgv-102-1622-s001.pdf]

FIG. S1 Aimone and Lavington et al

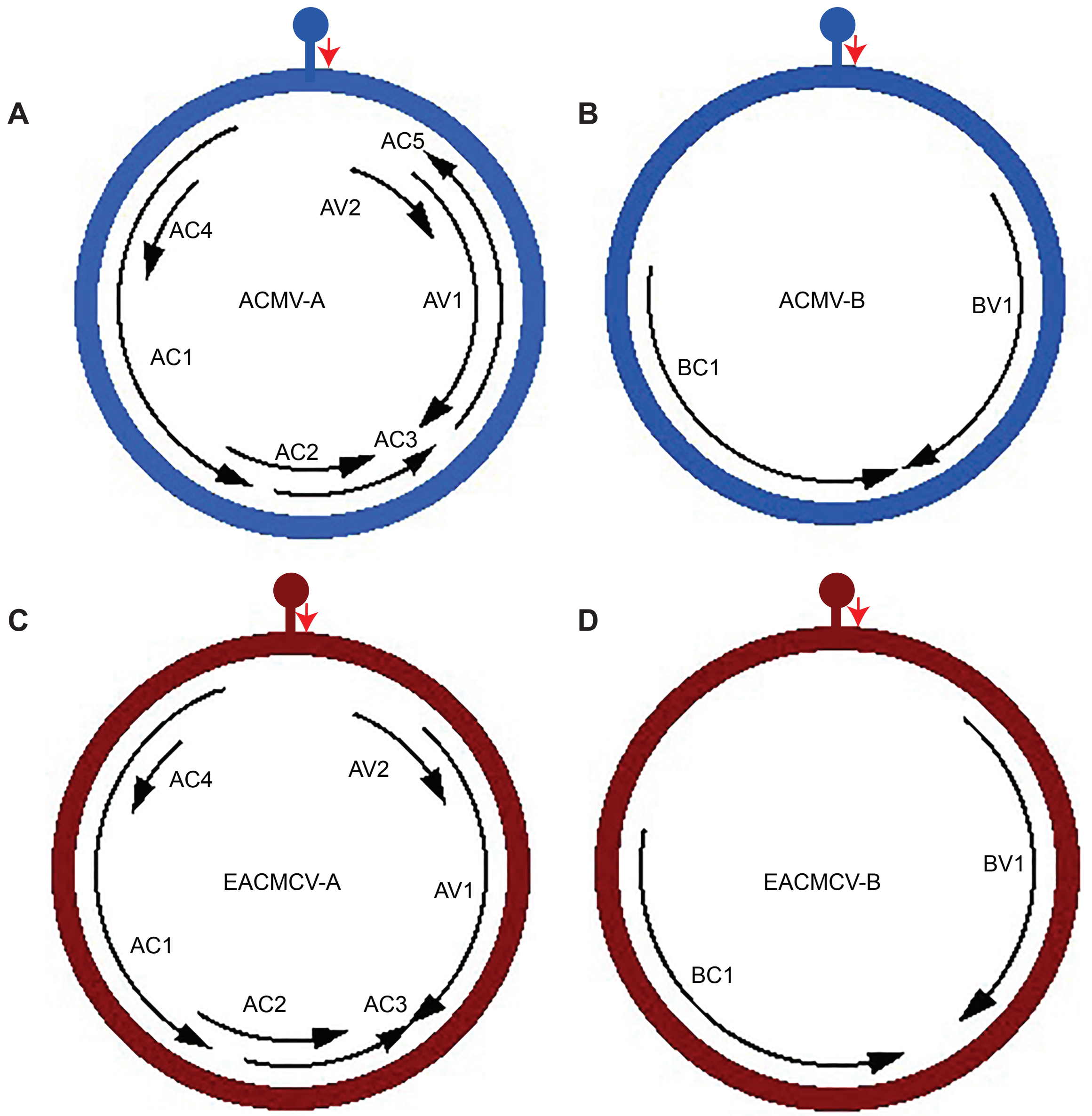

E

Gene Name	Protein Name	Major Function
AV1	CP	Mediates vector transmission
AV2	AV2	Anti-defense protein to inhibit post-transcriptional gene silencing (PTGS)
AC5	AC5	Counteracts TGS and viral replication
AC3	REn	Viral replication
AC2	TrAP	Interferes with transcriptional gene silencing (TGS) and PTGS
AC1	Rep	Viral replication
AC4	AC4	Counteracts TGS
BC1	MP	Movement of viral proteins out of the nucleus
BV1	NSP	Movement of viral particles from cell to cell

S1. ACMV and EACMCV clones. Diagram of A and B components of ACMV (a, b) and EACMCV (c, d). Black arrows represent opening reading frames, and the red arrow indicates nick site. (e) The table shows the gene name of each opening reading frame, the corresponding protein name, and function(s) of the protein in infection.

FIG. S2 Aimone and Lavington et al

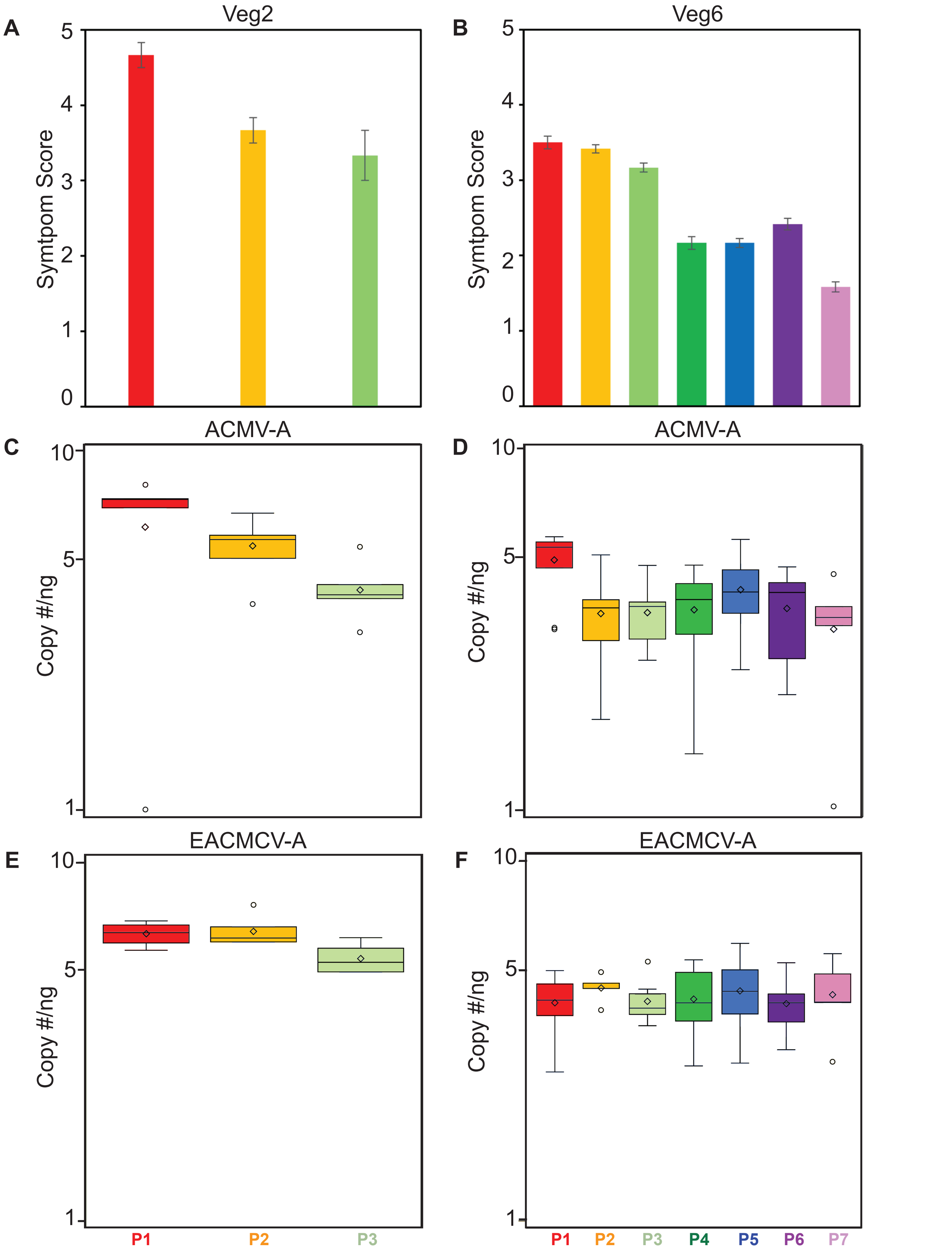

S2. Symptom and viral titer for Veg2 and Veg6. Veg2 (a) and Veg6 (b) experiments scored on a scale of 1 to 5 scale severity scale (scale: 1 = no symptoms to 5 = very severe) throughout new growth in the young leaves and leaf deformation at 28dpi. Log viral copy number/ng total DNA determined by qPCR of ACMV-A and EACMCV-A for Veg2 (S2c and e) and Veg6 (S2d and f), respectively.

FIG. S3 Aimone and Lavington et al

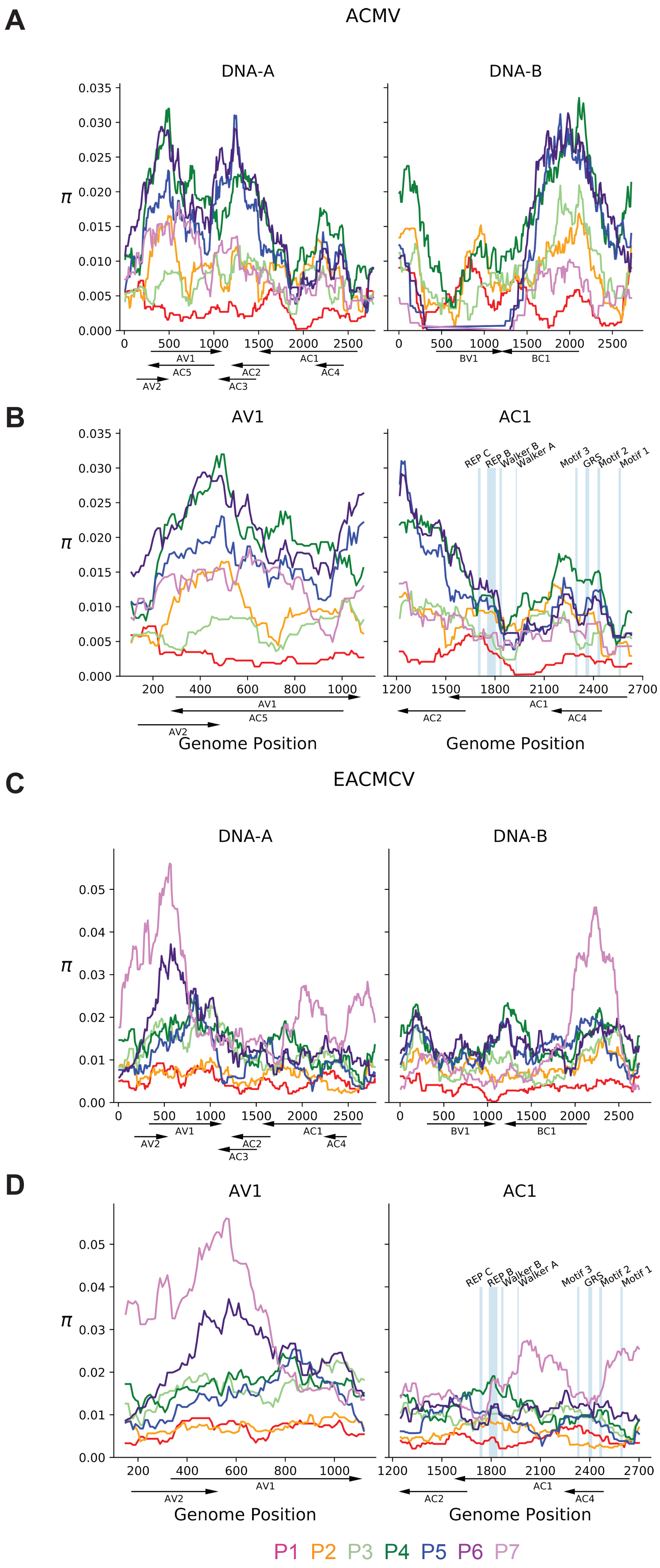

S3. Veg6 ACMV nucleotide diversity sliding windows of all 7 rounds. (a) Sliding window analysis of nucleotide diversity (pi) of ACMV DNA-A and DNA-B and (c) EACMCV DNA-A and DNA-B. Red to pink represents the nucleotide diversity across the genome of inoculated plants (P1) and six vegetative propagations (P2-P7). Enhanced views of the nucleotide diversity of the AV1 and AC1 open reading frames during P1-P7 for ACMV-A (b) and EACMCV-A (d). Blue lines mark the locations of codons encoding functional motifs in the Rep protein, i.e. Rep C, Rep B, Walker B, Walker A (63), Motif 3 (62), GRS (64), Motif 2 (62) and Motif 1 (83). The motifs are shown to scale. Genome coordinates (nt), the positions of open reading frames and their directions of transcription are shown below each graph.
